# Hybrid scheme for modeling local field potentials from point-neuron networks

**DOI:** 10.1186/1471-2202-16-S1-P67

**Published:** 2015-12-18

**Authors:** Espen Hagen, David Dahmen, Maria Stavrinou, Henrik Lindén, Tom Tetzlaff, Sacha van Albada, Sonja Grün, Markus Diesmann, Gaute T Einevoll

**Affiliations:** 1 Inst. of Neuroscience and Medicine (INM-6) and Inst. for Advanced Simulation (IAS-6), Jülich Research Center and JARA, Jülich, 52425, Germany; 2 Dept. of Mathematical Sciences and Technology, Norwegian University of Life Sciences, Aas, 1432, Norway; 3 Dept. of Neuroscience and Pharmacology, University of Copenhagen, Copenhagen, 2200, Denmark; 4 Dept. of Computational Biology, Royal Institute of Technology (KTH), Stockholm, 10044, Sweden; 5 Dept. of Biology, Theoretical Systems Neurobiology, RWTH Aachen University, Aachen, 52074, Germany; 6 Dept. of Psychiatry, Psychotherapy and Psychosomatics, Medical Faculty, RWTH Aachen University, Aachen, 52074, Germany; 7 Dept. of Physics, Faculty 1, RWTH Aachen University, Aachen, 52074, Germany; 8 Dept. of Physics, University of Oslo, Oslo, 0316, Norway

## 

**Figure 1:**
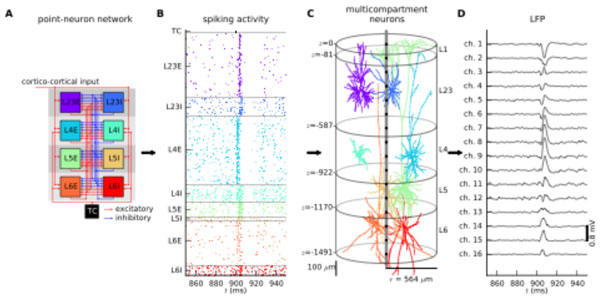
**Overview of the hybrid scheme for modeling LFP generated by a cortical network model**. **A **Sketch of point-neuron network model [1]. **B **Spikes of point neurons in the network for spontaneous and evoked activity. **C **Populations of multi-compartment neurons acting as LFP signal generators. **D **Depth-resolved LFP predicted using the hybrid scheme.

Measurement of the local field potential (LFP) has become routine for assessment of neuronal activity in neuroscientific and clinical applications, but its interpretation remains nontrivial. Understanding the LFP requires accounting for both anatomical and electrophysiological features of neurons near the recording electrode as well as the entire large-scale neuronal circuitry generating synaptic input to these cells. The direct simulation of LFPs in biophysically detailed network models is computationally daunting. Here, we instead propose a hybrid modeling scheme combining the efficiency of simplified point-neuron network models (Fig. 1A) with the biophysical principles underlying LFP generation by multicompartment neurons [1] (Fig 1C). We apply this scheme to a model representing a full-scale cortical network under about 1 square millimeter surface of cat primary visual cortex [2] (Fig. 1A,B) with layer-specific connectivity [3] to predict laminar LFPs (Fig. 1D) for different network states, assess the relative contribution of local neuron populations to the LFP, investigate the role of input correlations and neuron density, and validate linear LFP predictions based on population firing rates. The hybrid scheme is accompanied by our open-source software, **hybridLFPy **(github.com/espenhgn/hybridLFPy).
